# Space and time bisection in schizophrenia

**DOI:** 10.3389/fpsyg.2013.00823

**Published:** 2013-11-05

**Authors:** Isidro Martinez-Cascales, Juanma de la Fuente, Julio Santiago, Julio Santiago

**Affiliations:** ^1^Grounded Cognition Lab, Department of Experimental Psychology, University of GranadaGranada, Spain; ^2^Psychiatry Section, Hospital Dr. José Molina OrosaArrecife, Las Palmas, Spain

**Keywords:** common coding, schizophrenia, time, space, bisection tasks

## Abstract

As a test of the idea of a common brain system responsible for representing all prothetic dimensions, schizophrenic patients and healthy participants took part in a line bisection task and two visual temporal bisection tasks, one using durations from 1 to 4 s and another using 30 s long specially designed stimuli (aging faces). Against expectations, schizophrenics showed better precision (smaller variable error) both in line bisection and the aging faces temporal task than healthy controls. Moreover, patients also showed less bias (smaller constant error) than controls in the aging faces task. This increased precision correlated with degree of severity of schizophrenia. Although no group differences were found in the temporal task with shorter intervals, both variable and constant error measures correlated marginally with severity of schizophrenia, also in the direction of smaller error in more severe cases. Thus, overall, spatial and temporal tasks behaved similarly across groups. However, bias and precision indexes did not covary across the three tasks when correlations where computed over the whole set of participants in the present study. The results thus provide mixed support for a common system behind spatial and temporal processing and point toward the need of developing a more nuanced view of magnitude representation in the mind/brain.

## Introduction

How are abstract magnitudes, such as space, time, and numerosity, represented and processed? Do we use the same neuronal substrate for all dimensions? According to the highly influential A Theory of Magnitude (ATOM) proposed by Walsh (2003), prothetic dimensions (those which can be experienced as “more than” or “less than”), such as space, time, and numerosity, are processed by a common brain system located in the inferior parietal cortex. Views that share the basic assumption that disparate conceptual dimensions can have a common representational and processing substrate have been proposed also from other research traditions (see Lakoff and Johnson, 1980, for a linguistic analysis, and (Williams et al., 2009), from a social psychology standpoint). Although there are important theoretical differences between these perspectives, for current purposes we will subsume them all under the label of the “common coding hypothesis.”

Recent years have seen an increase in the interest on dimensional concepts (magnitudes such as space, time, pitch, evaluation, or social power; for reviews see Bueti and Walsh, 2009; Landau et al., 2010; Santiago et al., 2011; Bonato et al., 2012). In this context, a central research line focuses on the interactions between the processing of space and time (see Núñez and Cooperrider^2013^, for a recent review). For example, discriminating past from future times is faster if the “past” response is given with the left hand and the “future” response with the right hand than if the opposite mapping is used (Santiago et al., 2007). This pattern of interaction has most often been interpreted as revealing the use of a “mental time line,” a spatial representation underlying the processing of time. Analogous evidence have been used to support a spatial basis for understanding numerical magnitude (Dehaene et al., 1993), pitch (Rusconi et al., 2006), or social power (Schubert, 2005).

One important prediction of the common coding hypothesis is that the processing of different magnitudes should be associated, both in the performance of healthy adults as well as in the patterns of dysfunction in neuropsychological patients. So far, previous studies have found both evidence supporting this prediction (Bueti and Walsh, 2009; Bonato et al., 2012) and against it (Doricchi et al., 2005).

One way to address magnitude processing is by means of bisection tasks. These consist in estimating the central point of an interval in a given dimension. Classical examples are space and time bisection, joined more recently by number bisection tasks (e.g., Jewell and McCourt, 2000; Nuerk et al., 2002; Kopec and Brody, 2010). We will here focus on space and time bisection. In the most standard version of spatial bisection, line bisection, the participant is presented with a horizontal line which she has to mark at the center. The standard form of the time bisection task takes a different approach: the participant is presented with two reference (or anchor) stimuli which differ only in duration. In a learning phase the participant learns to discriminate the short and long stimuli. In a subsequent phase, stimuli varying in duration between the short and the long anchors are presented, and the participant judges whether they are closer to the short or long duration. The proportion of “long” responses is used to estimate the subjective middle point as well as the degree of discriminability or acuity. If all magnitudes somehow rely on a common representational system, their processing characteristics should covary. Thus, the prediction is that the bias and discriminability in spatial and temporal bisection tasks should covariate over tasks, groups, and individuals.

Schizophrenics have been reported to show different biases than healthy controls in the line bisection task. The picture, however, is complex. McCourt et al. (2008) replicated the well-known small leftward bias (pseudoneglect) usually observed in healthy participants (Jewell and McCourt, 2000) but found no bias in schizophrenic patients. Zivotofsky et al. (2007), in a study without a control group, replicated the schizophrenics' lack of bias in the line bisection task. In contrast, Michel et al. (2007) found a stronger leftward bias in patients than in healthy controls. Finally, Tian et al. (2011) also reported a stronger leftward bias in schizophrenic patients than healthy controls, who in this study did not show any bias. Thus, available studies show that both schizophrenic patients and healthy participants can sometimes bisect to the left or show no bias in line bisection. Their bisection biases are often different, and schizophrenics may sometimes bisect to the right, and sometimes to the left, of healthy participants.

Time perception, on the other hand, is generally agreed to be impaired in schizophrenia. Some studies have used time bisection tasks to address this temporal deficit. Carroll et al. (2008), using auditory and visual presentations of stimuli ranging from 300 to 600 ms, found that whereas both patients and controls were more accurate on auditory than visual intervals, schizophrenics were worse than controls only in the auditory modality. Carroll et al. (2009a), using only auditory stimuli, replicated the reduced temporal acuity in the 300–600 ms range and extended it to the 3000–6000 ms range. Lee et al. (2009) also found decreased accuracy in the discrimination of auditory durations both in the 400–800 and 1000–2000 ranges, and they also observed a fixed bias toward underestimating intervals in the 400–800 condition (that is, a greater proportion of “short” responses, leading to a subjective middle shifted toward the “long” duration). Finally, Elvevåg et al. (2003) also found decreased temporal precision with auditory stimuli between 200 and 800 ms, plus a constant bias which again took the form of interval underestimation. Thus, available evidence suggests that schizophrenics have a deficit in time perception in the auditory modality that spans intervals in the range from 200 to 6000 ms. There is also some evidence for a constant bias toward perceiving sub-second intervals as shorter than they actually are, although this bias has not always been replicated. However, their visual time bisection might be spared.

Other time perception tasks have also shown temporal deficits in schizophrenia. When tapping to a rhythm of 2 tones per second and then trying to keep the same rhythm without external guidance, schizophrenics show increased variability and a tendency to shorten the intervals (Carroll et al., 2009b). Patients also show more errors than controls when comparing a subsequent auditory duration (range 310–490 ms) to a prior standard duration (400 ms). Tasks assessing longer intervals, in the range of tens of seconds, have typically asked patients to either stop an interval at a specified duration (say, after 20 s), or to estimate verbally the duration of an interval in seconds. Common findings are: (1) a tendency to overestimate intervals (Tysk, 1983), which runs contrary to the bias toward shorter intervals discussed above; (2) markedly different individual patterns, with some patients being clear underestimators and other overestimators (Tysk, 1984); and (3) a lack of clear differences with healthy controls (e.g., Tracy et al., 1998). Given that these longer intervals are often unfilled, these tasks are open to the use of strategies (mainly, counting) and to the effects of boredom and distraction, and so they may reflect attentional deficits more than proper temporal processing.

The aim of the present work was to shed light on the relationship between the processing of the magnitudes of space and time by studying performance on bisection tasks in a sample of healthy participants (Experiment 1), and a group of schizophrenic patients and a matched control group (Experiment 2). In the temporal dimension, we decided to explore visual durations in the supra-second range, as these have been understudied using bisection tasks in schizophrenia (see above). We used both a standard time bisection task with stimuli ranging from 1 to 4 s and a novel task using 30 s long stimuli specially designed to avoid problems of lack of motivation, boredom, distraction, as well as counting and other idiosincratic strategies. By doing so, we hoped to extend current knowledge about time processing in schizophrenics. Moreover, finding similarities in bias or discriminability across dimensions would argue in favor of a common coding of magnitude. Conversely, no evidence of covariation between tasks in the different groups of participants would suggest independent underlying systems for each magnitude.

## Experiment 1: space and time in the general population

In this first experiment we wanted to test whether we could find linked biases in space and time bisection in the general population. We measured indexes of both bias and precision in one spatial and two temporal bisection tasks, as well as their correlation over individuals.

To measure spatial bisection we used a Line Bisection task: lines were presented to be bisected at the center. Regarding temporal bisection, we used two tasks: a Standard Time Bisection task, as described above, and a novel temporal bisection task using human faces that age progressively over a total interval of 30 s: the Aging Faces task. These stimuli have the advantage of capturing the attention of the perceiver to the stimulus face as it changes from being a child to an elderly person, thereby avoiding distraction and boredom. After the stimulus clip, frames extracted from it at regular intervals are presented to be judged as closer to the beginning or the end of the video, and the proportion of “end” responses to each frame is analyzed in analogous fashion to the Standard Time Bisection task.

The Aging Faces task, as used here, is adapted from tasks which have already been used to study temporal processing in prior studies. Santiago et al. (2010; Experiment 1) presented their participants with video clips extracted from commercial movies. After watching the clip, participants saw frames from the clip and produced left and right keypresses to indicate whether the frame belonged to the first or second half of the clip. Subsequent experiments in that series used analogous tasks, using sequences of six pictures depicting everyday events (e.g., preparing breakfast) instead of video clips. A similar approach was used by Fuhrman and Boroditsky (2010) and Fuhrman et al. (2011) using sequences of three pictures such as the face of Julia Roberts at different ages, a banana being eaten, and so on. In all these studies, the results showed the well-known congruency effect between space and time (Santiago et al., 2007): participants were faster to respond when “beginning” or “past” was mapped to the left hand and “end” or “future” to the right hand than when using the opposite mapping. The Aging Faces task builds on those procedures but without the use of lateralized manual responses, in order to avoid any induction of the use of a spatial representation. The data are analyzed in a way that allows the assessment of psychophysical properties of the underlying representation, such as their subjective midpoint and its perceptual acuity.

### Method

#### Participants

Twenty five (5 male, one left hander, average 23.8 years, range 18–49) students from the psychology degree at University of Granada took part in this experiment. They received course credit for their participation.

#### Tasks

***Line bisection***. Participants were asked to mark with a pen the middle point of a horizontal line (1 mm wide, 200 mm long) printed centered on a sheet of paper. After the response, the sheet was removed from the desk and another identical sheet was presented. Each participant bisected a total of 24 lines.

All lines from each participant were then scanned and the location of the mark was measured in millimitres using a graphics processing program (precision: ±0.1 mm). The distance between the mark and the actual center was then measured and used to compute the average bias and its standard deviation for each participant. Average bias was the index of constant bias, and standard deviation was the index of precision.

***Standard time bisection task***. The stimulus was a blue square (6.8 × 6.8 degrees of visual angle) presented at the center of a 15″ screen monitor for a duration ranging between 1000 and 4000 ms. Participants first received a training block of 20 trials to learn to discriminate between the two extreme (or anchor) durations, 1000–4000 ms. Only the two anchors were presented in this block, 10 times each one in random order. All participants learnt to discriminate the anchors during training (final accuracy range 95–100%). Next, they received 154 trials where the durations 1000, 1500, 2000, 2500, 3000, 3500, and 4000 ms were randomly presented. Participants were asked to estimate, for each stimulus, whether its duration was closer to the short or long anchor. The two anchors appeared three times more often than the intermediate durations in order to keep refreshing them throughout the task. Responses were given verbally. In order to prevent any spatial activation generated by the action of responding, the experimenter coded them simultaneously by means of keypresses out of the participant's sight. Stimulus duration was converted to a continuous scale from 0, the shortest duration, to 10, the longest duration, and the proportion of “long” responses for each stimulus duration was computed for each participant. These data typically took the shape of a cumulative curve. The curve was then fitted to a linear model. The slope and intercept of this line were used to calculate the Point of Subjective Equality (PSE) and the Just Noticeable Difference (JND) for each participant (PSS = −intercept/slope, which is mathematically equivalent to the stimulus duration at which the best fitted line crosses the 0.5 proportion; JND = 0.675/slope, which corresponds to substracting the stimulus duration at which the function crosses the 0.75 proportion from the duration at which the same function crosses the 0.25 proportion and then dividing it by two; see Coren et al., 1999). The PSE indexes the subjective midpoint of the temporal interval between the two anchors and allows the assessment of constant bias. The JND estimates the minimum distance between two points in the interval that the participant is able to recognize as different, and thus it serves as an index of perceptual acuity. A high JND score indicates a high variability in the responses (low acuity) whereas a low JND indicates the ability to discriminate smaller intervals. In this and the following task, Eprime 2.0 software (Psychology Software Tools Inc.) was used for stimulus presentation and data collection.

**Figure 1:**
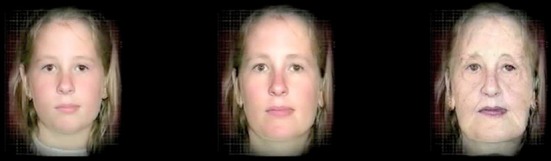
**Three frames extracted from initial, middle, and final moments in one of the video clips of the Aging Faces task**.

***Aging faces task***. Participants saw four videos, each showing a face (two male, two female) that aged gradually from childhood to elderly. Videos were extracted from a demo videoclip of the April Age Progression software (downloaded from Youtube - https://www.youtube.com/watch?v=fa5rzZroNyU). Each clip lasted for 30 s, and was presented twice. Figure 1 shows three frames of one of them as an example. Following the second presentation of a clip, 36 frames extracted at constant intervals throughout the clip were presented in random order. Each frame was presented three times, totalling 108 trials per clip. Participants estimated whether the frame was temporally closer to the beginning or to the end of the clip. As in the previous task, responses were given verbally and coded online by the experimenter through hidden keypresses. After all frames on a given video clip were presented, the procedure was repeated for the next video. Data from this task were analyzed following an analogous procedure to Standard Time Bisection, rendering PSE and JND indexes.

Summing up, bias indexes include the average distance from the mark to the actual center of the line in the Line Bisection task, and the PSE in the Standard Time Bisection and Aging Faces tasks. Henceforth, these will be referred to as “bias.” Precision indexes include the standard deviation of the distance of the marks to the actual center in Line Bisection, and the JND in the two temporal tasks. These will be referred to as “precision.”

#### Procedure

Participants were tested individually in a single session. Task order was kept constant throughout the experiment. The Standard Time Bisection task was presented first, followed by the Aging Faces task and, finally, the Line Bisection task. Temporal bisections were administered before Line Bisection in order to prevent a preactivation of the spatial dimension in the temporal tasks.

### Results

**Figure 2:**
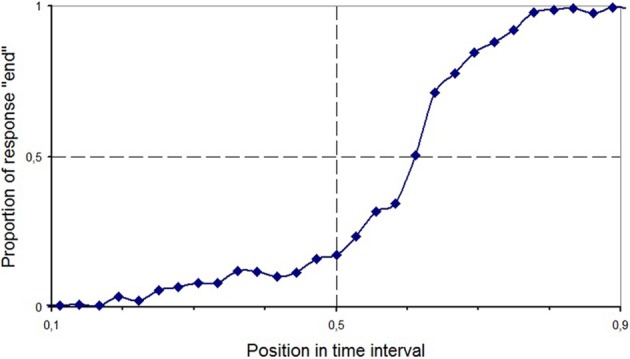
**Proportion of trials with “end” response as a function of temporal position in the video clip in the Aging Faces task in Experiment 1 (healthy participants)**.

The bias indexes were analyzed by means of two-tailed *t*-tests against the central value for each task. No significant bias was found neither in Line Bisection [*t*_(24)_ = −0.33, *p* = 0.74] nor in the Standard Time Bisection task [*t*_(24)_ = −0.06, *p* = 0.95]. However, in the Aging Faces task, data showed that the PSE was displaced toward the end of the interval [*t*_(24)_ = 3.43, *p* = 0.002; see Figure 2]. In other words, participants produced more “beginning” than “end” responses.

Importantly for our hypotheses, we tested whether the indexes of bias and precision covaried over participants across the dimensions of space and time by means of Pearson's correlation coefficients. All correlations between spatial and temporal tasks were close to zero and non-significant. The two temporal tasks were also not correlated to each other, what suggests that they tap onto different temporal mechanisms.

### Discussion

We failed to replicate the pseudoneglect effect in the Line Bisection task. This is not entirely surprising, given that it is not a very robust effect and it can be affected by a variety of factors (see Jewell and McCourt, 2000). The end bias (the tendency to locate the midpoint of the interval closer to the end) found in the Aging Faces task is also difficult to interpret. Although the instructions explicitly asked participants to decide whether a given frame was closer to the beginning or the end of the video, participants may have interpreted the task as deciding whether a picture of a person is closer to the beginning or the end of his or her life. This subjective vital midpoint may not have coincided exactly with the midpoint of the clip. As it is likely that what a participant considers to be the midpoint of somebody else's life is linked to what she takes to be the midpoint of her own life, and because this probably varies depending on participant's age, Experiment 2, where we collected data from older participants, will help clarifying this point.

The main result of the first study is a lack of evidence supporting a link between space and time processing in the general population, as shown by null correlations across tasks. However, the absence of cross-task correlations in one single group does not constitute strong evidence against the hypothesis of common coding. In our second experiment, we searched for a relationship between space and time in a group of schizophrenic patients, whose spatial and temporal processing is known to differ from the general population.

## Experiment 2: schizophrenic patients and matched healthy controls

### Method

#### Participants

Twelve schizophrenic patients (five female, one left hander) and a control group of 12 participants with no previous psychiatric diagnosis (four female, one left hander) gave their consent to participate in this study (see Table 1). The two groups were matched in age [*t*_(22)_ = 1.57, *p* = 0.26] and educational level [*t*_(22)_ = −0.42, *p* = 0.68]. Both groups had zero experience in participating in psychology experiments or taking computerized tests, and they all had very low experience with computers generally. Participants received a small gift for their cooperation. All participants in the schizophrenic group were outpatients who met the DSM-IV criteria for schizophrenia, were clinically stable, and were taking medication at the time of study.

**Table 1:** **Demographic and clinical variables of participants in Experiment 1**.

**Group**	**Gender**	**Age**	**Handedness**	**Educ.**	**DSM IV**	**DSM IV code code**	**PANSS Pos.**	**PANSSNeg.**	**PANSSGen. Psych.**	**PANSS Total**	**Severity Rank**	**GAF**	**Chl. Equiv.**	**Medication**
Control	Female	36	Right	13	–	–	–	–	–		–	–		–
Control	Male	22	Right	12	–	–	–	–	–		–	–		–
Control	Male	40	Right	8	–	–	–	–	–		–	–		–
Control	Male	27	Right	8	–	–	–	–	–		–	–		–
Control	Female	35	Right	13	–	–	–	–	–		–	–		–
Control	Male	30	Left	12	–	–	–	–	–		–	–		–
Control	Female	43	Right	12	–	–	–	–	–		–	–		–
Control	Male	31	Right	8	–	–	–	–	–		–	–		–
Control	Male	40	Right	8	–	–	–	–	–		–	–		–
Control	Male	45	Right	8	–	–	–	–	–		–	–		–
Control	Male	28	Right	9	–	–	–	–	–		–	–		–
Control	Female	25	Right	9	–	–	–	–	–		–	–		–
Patient	Female	36	Right	12	Schizophrenia—paranoid	295.30	11	16	43	70	12	50	861	Paliperidone, Ziprasidone, Risperidone
Patient	Female	32	Right	8	Schizophrenia—paranoid	295.30	24	26	44	93	7	42	100	Topiramate, Levomepromazine maleato
Patient	Male	25	Right	6	Schizophrenia—paranoid, mental retardation	295.30, 317	16	24	39	79	11	44	1033	Paliperidone, Amisulpride, Levomepromazine maleato
Patient	Male	30	Right	5	Schizophrenia—paranoid	295.30	17	31	42	90	10	46	700	Amisulpride, Levomepromazine maleato
Patient	Male	45	Right	12	Schizophrenia—paranoid	295.30	25	18	39	82	5	52	456	Zuclopenthixol decanoate, Ziprasidone
Patient	Male	48	Right	7	Schizophrenia—paranoid	295.30	24	22	34	80	6	50	220	Paliperidone
Patient	Male	45	Right	8	Schizophrenia—paranoid	295.30	23	23	32	78	9	36	596	Risperidone, Fluphenazine decanoate
Patient	Female	38	Right	12	Schizoafective disorder	295.70	19	21	45	85	3	52	0	Lithium carbonate, Escitalopram oxalate
Patient	Female	33	Left	12	Schizophrenia—disorganized	295.10	22	19	38	79	4	44	775	Quetiapine fumarate, Ziprasidone
Patient	Male	39	Right	11	Schizophrenia—paranoid	295.30	14	19	30	63	1	50	376	Ziprasidone, Clozapine
Patient	Female	31	Right	10	Schizoafective disorder	295.70	14	19	28	61	8	48	800	Clotiapine, Clonazepam
Patient	Male	41	Right	12	Schizophrenia—paranoid	295.30	19	20	34	73	2	56	1067	Clozapine, Amisulpride, Aripripazole

Education level is coded as number of schooling years since age 6. For each patient, the following information is presented: (1) scores obtained in the three subscales (Positive, Negative, General Psychopathology) of the Positive and Negative Syndrome Scale (Kay et al., 1987), as well as its total score; (2) the Global Assessment of Functioning (GAF; DSM-IV-TR, 2000); (3) the Severity Rank (see description in main text); (4) Medication, in DCI names; and (5) Chlorpromazine equivalents for the medication and dosage of each patient. The following conversion rates were used (in mgr/day equivalents to a dosis of 100 mgr/day of chlorpromazine): Amisulpride (100)^a^, Aripripazole (7.5)^a^, Clotiapine (10)^b^, Clozapine (100)^a^, Fluphenazine decanoate (2)^c^, Levomepromazine maleato (100)^a^, Paliperidone (1.5)^d^, Quetiapine fumarate (77)^a^, Risperidone (1.5)^a^, Ziprasidone (62.5)^a^, Zuclopenthixol decanoate (14.3)^a^. The superscript in each drug refers to the source where equivalent doses were obtained from: (a) Kroken et al. (2009); (b) Tibaldi et al. (1997); (c) Atkins et al. (1997); (d) Woods (2011).

For each patient, three severity indexes were considered in the following analyses. The first was the Global Assessment of Functioning (GAF), presented and described in the DSM-IV-TR (2000), which ranges from 1 to 100 (being 100 the best functioning individuals). Because all patients had very similar GAF assessments, we also used two more severity indexes. A second index consisted on a ranking (from 1 to 12) of the patients from the less severe to the most severe case. This ranking was made by one of the authors (Julio Santiago Sr.), who has treated all of the patients in this study since the inception of their illness (in all cases over many years). The judge was asked to rank all patients depending on their severity, taking into account the severity of the schizophrenic symptoms, degree of cognitive deterioration, family support and ability to adapt in society (as we wanted to capture the overall impression of severity from a specialist who deeply knows each case in all its dimensions, no specific weighting of these factors was suggested, and it is possible that they were weighted differently in different cases). These are all factors that the GAF takes into account, but this index (which we will call the Severity Ranking henceforth) allowed us to make finer distinctions within the group of patients. Crucially, the ranking was made without knowledge of the individual results of the patients in the experimental tasks. A final index, the Chlorpromazine Index, was based on the dosis of neuroleptic medication being administered to each patient at the moment of the study. In order to convert the different drugs to a common scale, neuroleptic doses were converted to Chlorpromazine equivalentes, and when the prescription included more than one neuroleptic, their Chlorpromazine equivalents were added up (see Table 1 for details and references). The Chlorpromazine Index is a rough proxy to severity, because different neuroleptics (specially since the introduction of atypical neuroleptics) vary in their affinities for different receptors, each receptor mediating different cognitive functions, and therefore, their effects do not line up along a single dimension. However, it is still widely used in clinical research on schizophrenia^1^. For the sake of data analysis we assigned the control participants a score of 90 in GAF, 0 in the Severity Ranking, and 0 in the Chlorpromazine Index.

#### Tasks

We used the same three tasks as in Experiment 1: Line Bisection, Standard Time Bisection and Aging Faces temporal bisection. The number of trials in Standard Time Bisection was reduced in order to make it easier for schizophrenic participants to keep a steady level of attention throughout the experiment. In this version of the task, after 20 trials of practice with the anchors, each level of duration (1000, 1500, 2000, 2500, 3000, 3500, and 4000 ms) was presented 12 times, totalling 84 trials.

#### Procedure

Participants were tested individually. The three tasks were presented sequentially, always in the same order, with a break after each one. The pace of all tasks was adapted to each participant. Most participants (11 patients and 9 controls) did the experiment in two sessions separated by at least 24 h. The first task was the Aging Faces task, followed by Line Bisection if the participant was willing to continue. The second session consisted of the Standard Time Bisection task followed by Line Bisection if it had not previously been done. Both temporal tasks were therefore kept apart for most participants, and the spatial task was always presented last in a session, in order to prevent a preactivation of the spatial dimension in the temporal tasks. Participants who finished the experiment in a single session did Aging Faces first, followed by Standard Time Bisection, and finally Line Bisection.

### Results

Deviations from normality were tested using the Kolmogorov-Smirnov test and associated Lilliefors probabilities. In spite of the small sample sizes, data in all indexes in both groups did not differ significantly from a normal distribution with the only exception of the precision index (JND) of the Aging Faces task in the healthy control group (Kolmogorov-Smirnov *d* = 0.25, Lilliefors *p* < 0.05). Therefore, comparisons involving this index in the healthy control group were also carried out using non-parametric alternatives. When normality was assessed pooling together controls and patients, or controls and the younger healthy participants from Experiment 1, no index deviated from normality, and therefore the use of parametric correlations over the pooled samples was justified.

As in Experiment 1, two-tailed *t*-tests showed no deviation from the actual midpoint in Line Bisection and Standard Time Bisection, both pooling together all participants as well as taking each group independently (in all cases, *p* > 0.22). In the Aging Faces task we again found a significant bias toward the “end” side of the time interval, but only in the control group [*t*_(11)_ = 5.79, *p* = 0.0001], and not in the schizophrenic group [*t*_(11)_ = 1.73, *p* = 0.11]. The difference in bias between the groups was significant [*t*_(22)_ = 2.31, *p* = 0.03]. Thus, schizophrenic patients showed a null bias whereas control participants produced a greater proportion of “beginning” responses, leading to a displacement of the subjective middle toward the end of the interval. This difference cannot be due to age, educational level, familiarity with computers or computerized testing, as both groups were matched in these variables.

As discussed in Experiment 1, one possible explanation for the end bias in the Aging Faces task is that participants are estimating a vital midpoint, that is, the midpoint of the life of the depicted person, instead of the midpoint of the duration of the videoclip. Assuming that the estimation of someone's vital midpoint is linked to the perceiver's own estimated vital midpoint, and assuming that people tend to think that they are still in the first half of their lives (and thus that they are still young), a prediction from this account is that the subjective vital midpoint should be affected by the age of the participant: older participants should show a greater end bias. Put in other words, older participants should judge people of more advanced age as still closer to the beginning than to the end of their lifes. In order to test this prediction, we compared the PSE in the healthy control group in Experiment 2 (mean age 33.5 years) to the younger healthy participants in Experiment 1 (mean age 23.8 years). The two groups were indeed different [*t*_(35)_ = 2.17, *p* = 0.04]: the older group showed a greater end bias (i.e., a greater tendency to produce a “beginning” response). However, the PSE did not correlate with age in the total sample of healthy participants (*r* = 0.08). Looking only at schizophrenic patients, the correlation between PSE and age was also close to null (*r* = −0.02). In other words, older participants did not estimate that the midpoint of an aging sequence is farther along the life. Therefore, the data do not support the suggestion that participants are estimating a vital midpoint in the Aging Faces task instead of the actual midpoint of the temporal interval comprised by the beginning and end of the videoclip. The difference in bias in the Aging Faces task between the two groups of healthy participants must be due to any of the other factors that distinguish them (e.g., level of education, experience with computerized testing). A similar contrast in the PSE in the Standard Time Bisection task found no difference between the two healthy groups [*t*_(35)_ = −0.41, *p* = 0.68], and so the difference seems to be specific to the Aging Faces task. At any rate, it must be emphasized that whatever is causing the bias toward the end of the video clip in the healthy groups, the fact still remains of a smaller bias in the schizophrenic group when compared to a properly matched control group. In other words, schizophrenics are not affected by whatever factor is biasing the constant error of healthy controls.

Additionally, control and schizophrenic groups also differed in the precision of their performance, marginally in Line Bisection [*t*_(22)_ = −0.2, *p* = 0.056] and clearly in Aging Faces [*t*_(22)_ = −3.66, *p* = 0.001; Mann-Whitney *U* = 15, *z* = −3.29, *p* = 0.001]. Surprisingly, schizophrenic patients were more precise than matched healthy controls in both tasks. In contrast, the Standard Time Bisection task did not show any difference between groups [*t*_(22)_ = −0.77, *p* = 0.45].

**Figure 3:**
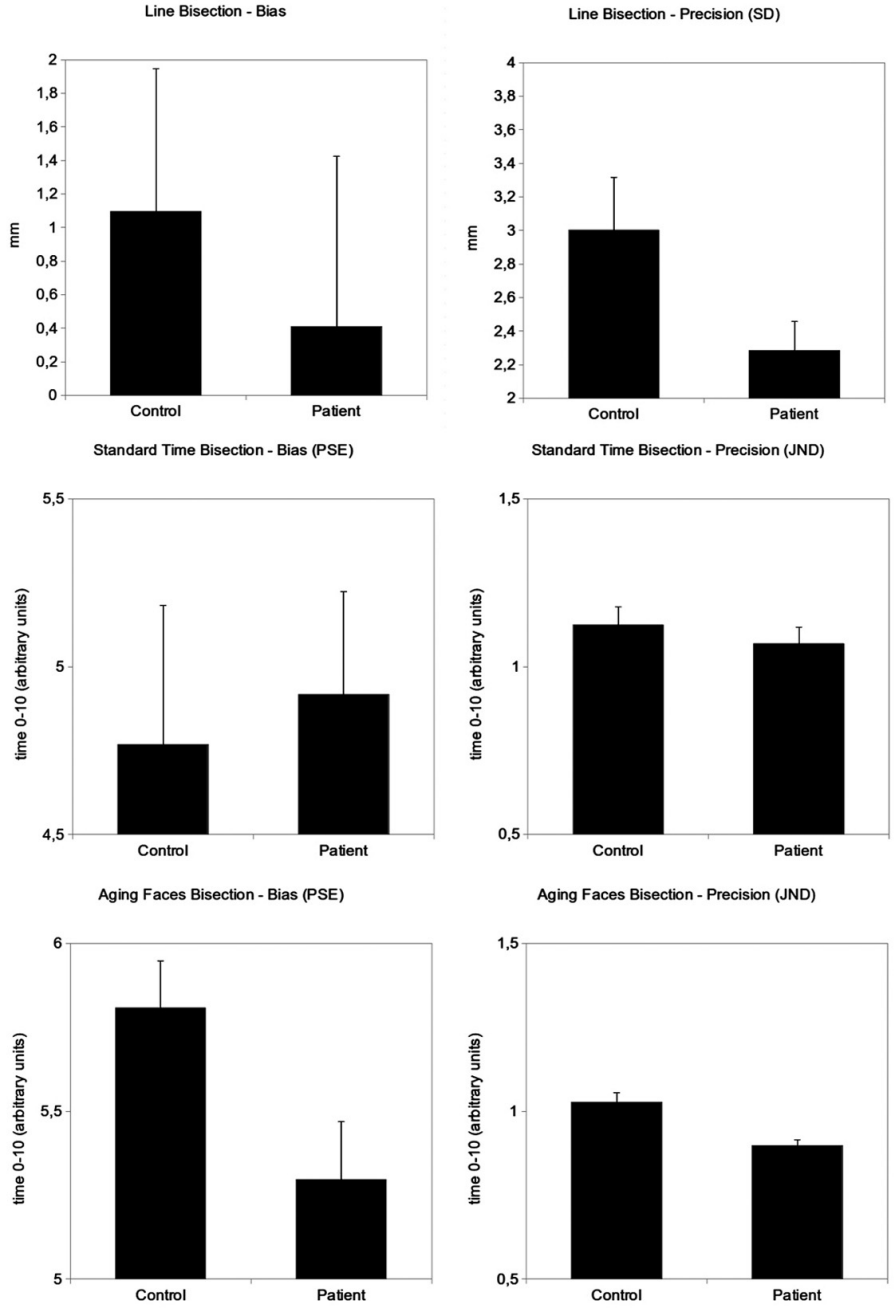
**Bias and precision indexes in one spatial (Line Bisection) and two temporal (Standard Time Bisection and Aging Faces Bisection) tasks.** Bias (constant error) and precision (standard deviation) in Line Bisection are expressed in millimetres. Bias (PSE) and precision (JND) in both the Standard Time Bisection and the Aging Faces tasks are expressed in arbitrary units from 0 to 10 where 0 is the beginning and 10 the end of the stimulus. Error bars represent standard error of the mean.

To sum up, schizophrenic patients showed a null bias in the Aging Faces task, in contrast to the significant end bias shown by controls. They also showed greater precision in both Line Bisection and Aging Faces, but not in Standard Time Bisection. Figure 3 shows these results.

Results from cross-group comparisons are further supported as well as qualified by correlation analyses with severity of schizophrenia. When data from the whole set of participants were used, three performance indexes correlated significantly to the Severity Ranking: precision in Line Bisection (*r* = −0.51, *p* = 0.01), and both bias and precision in Aging Faces (bias: *r* = −0.56, *p* = 0.005; precision: *r* = −0.42, *p* = 0.04). The Chlorpromazine Index also correlated significantly with precision in Line Bisection (*r* = −0.50, *p* = 0.014), and both bias and precision in Aging Faces (bias: *r* = −0.61, *p* = 0.002; precision: *r* = −0.54, *p* = 0.007). These results support the finding of greater precision in both space and one temporal task (Aging Faces) in the schizophrenic group that were revealed by cross-group comparisons. The GAF index of severity was less sensitive (as expected from its smaller variability within the schizophrenic group) and only correlated significantly to precision in Aging Faces (*r* = 0.62, *p* = 0.001). When only data from the schizophrenic group were used, reducing sample size to half, the correlations between both the Severity Ranking (*r* = −0.74, *p* = 0.006) and the Chlorpromazine Index (*r* = −0.68, *p* = 0.016) to precision in Line Bisection remained significant. Moreover, the Chlorpromazine Index also correlated significantly with bias in the Aging Faces task (*r* = −0.61, *p* = 0.035).

**Figure 4:**
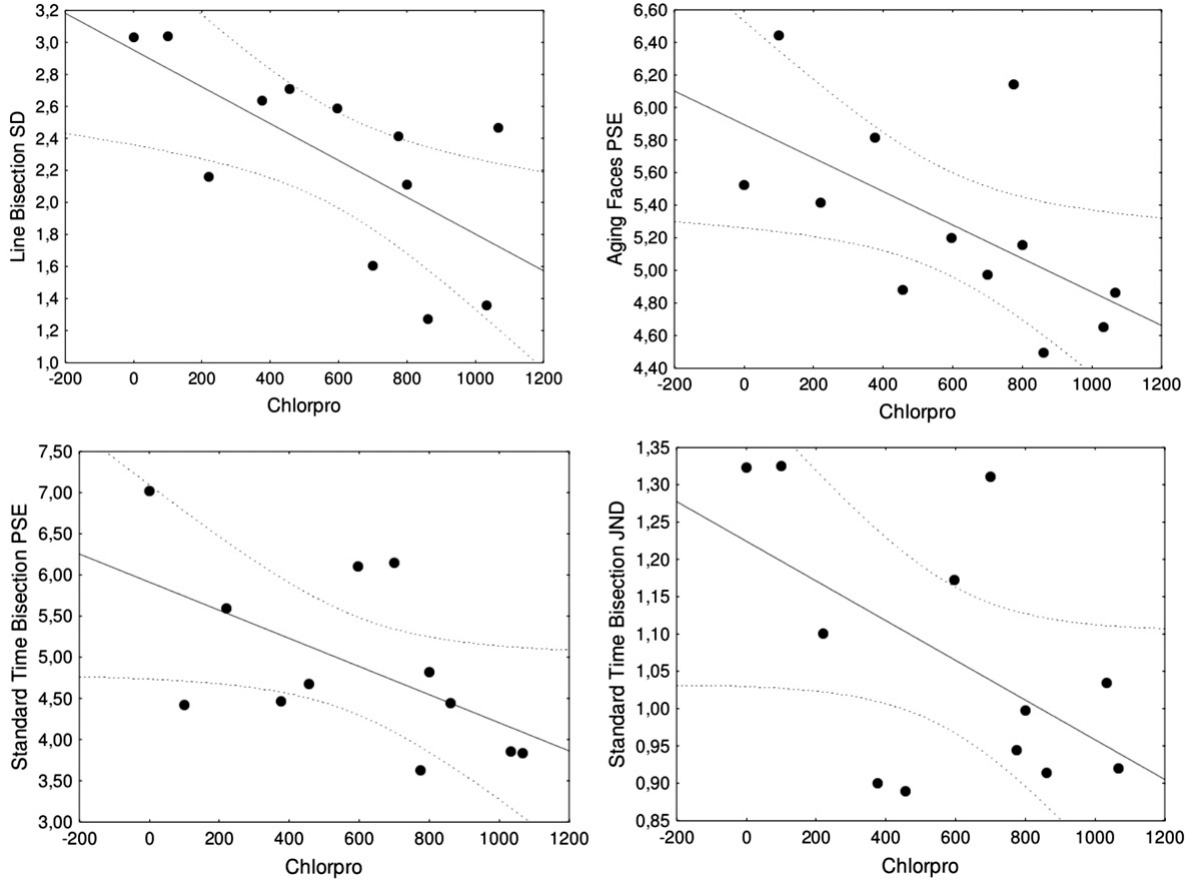
**Significant and marginal correlations including only the group of schizophrenic patients between the Chlorpromazine index (Chlorpro) and performance indexes, with best-fitting regression line and 95% confidence intervals.** The two upper scatterplots are significant correlations with precision in Line Bisection (left) and bias in Aging Faces (right). The two lower scatterplots are marginally significant correlations with bias in Standard Time Bisection (left) and precision in Standard Time Bisection (right).

Qualifying the prior finding of no differences between groups in the Standard Time Bisection task, the correlation between the Chlorpromazine Index and precision in the Standard Time Bisection task computed pooling together patients and controls approached reliability (*r* = −0.36, *p* = 0.087). When only data from the schizophrenic group were used, the Chlorpromazine Index showed marginally significant correlations with both performance indexes in the Standard Time Bisection task (bias: *r* = −0.57, *p* = 0.055; precision: *r* = −0.54, *p* = 0.067). Both error indexes (bias and precision) decreased with severity. Figure 4 shows scatterplots of the significant and marginal correlations between the Chlorpromazine index and performance indexes in all tasks, which suggest that these results are not due to a few outliers.

Correlations with severity of schizophrenia should be taken only as additional supportive evidence for the main results, revealed by cross-group comparisons. Their utility is limited, first, by the small sample size and the small variability in severity, as all cases were quite severe cases. Secondly, the two most sensitive indexes of severity (the Severity Ranking and the Chlorpromazine Index) suffer from potential problems. The Severity Ranking is based on the opinion of a single (though highly expert) judge. Because these ratings were made blind to the performance of the patients in the experimental tasks, the expectations of the judge are unlikely to have introduced any bias in the results, but ideally these ratings should have been obtained from at least two independent judges and their agreement computed. Unfortunately, it was not possible to have a second expert judge. In turn, the Chlorpromazine Index is based on a blend into a single scale of the affinities of different neuroleptic drugs for different receptors in the brain, which in turn mediate a variety of cognitive functions. With these cautions in mind, correlations of the performance indexes in one spatial and two temporal bisection tasks with the three indexes of severity (GAF, Severity Ranking, and Chlorpromazine Index) supported the main findings of the cross-group comparisons: Schizophrenic patients showed greater precision than matched controls in both Line Bisection and Aging Faces, as well as a smaller bias in Aging Faces. They also left open the possibility that severity of schizophrenia could be linked to both bias and precision in the Standard Time Bisection task. As this final finding was not supported by cross-group comparisons and it only approached conventional significance levels in the correlation analysis, we suggest caution in its interpretation.

In order to test whether there is a common system underlying the processing of different magnitudes, we analyzed the correlations between the indexes in spatial and temporal tasks, first for bias and then for precision. There were no significant correlations between the bias indexes in the three tasks. Regarding precision, Line Bisection correlated with Standard Time Bisection (*r* = 0.44, *p* = 0.03) and Aging Faces (*r* = 0.49, *p* = 0.016). Thus, greater precision in line bisection covaried with greater precision in each of the two temporal tasks. However, those two correlations turned non-significant when each group was considered separately. Most importantly, they remained non-significant when computed over the total set of participants in Experiments 1 and 2, what supports the conclusion that there was no cross-task covariation. The correlation between precision in the two temporal tasks, Standard Time Bisection and Aging Faces, was also not significant, again suggesting that the two tasks tap onto different temporal mechanisms.

### Discussion

As in Experiment 1, we found no pseudoneglect in Line Bisection, neither in the control nor schizophrenic groups. Also similarly to Experiment 1, we found a bias toward the “end” side of the time interval in the Aging Faces task, but this time only in controls: Schizophrenic patients showed no bias. The end bias in the Aging Faces task in the control group was stronger than in the younger participants of Experiment 1, but it did not correlate with participant's age. It is possible that a sample with a greater age range may show a significant effect of age on this bias, but present data do not support this link, and therefore neither they support the possibility that participants are bisecting a life-long vital interval instead of the clip duration. Future research is necessary to isolate the relevant factor that differentiates the two healthy groups, as they also differed in several other factors (e.g., education level or computer literacy). Whatever the cause, there remains the fact that schizophrenic patients bisected temporal intervals in the Aging Faces task closer to the actual center (actually showing no bias) than properly matched controls.

We also found evidence that schizophrenic patients showed *greater* precision than matched controls in both the spatial Line Bisection task and the temporal Aging Faces task, suggesting a three-way link between schizophrenia, space, and time. In the Aging Faces task, test faces are chosen from the videoclip and presented in random order to be judged regarding their proximity to the beginning or the end of the clip. Thus, the task is a demanding task and the fact that patients are performing with greater temporal acuity than controls, instead of showing a decrement in performance, argues against any deficit of a general nature that could affect cognitive processing across the board. They also argue agains the possibility that patients' performance is a result of medication, as medication should produce deficits in performance. Moreover, the specific link between schizophrenia and space (Line Bisection) and schizophrenia and time (Aging Faces) was supported by correlation analyses, which showed that as both severity and dosage increased, some of the indexes of performance improved.

The Standard Time Bisection task did not discriminate between patient and control groups, although it marginally correlated with severity of schizophrenia in one of the indexes, leaving open the question of whether schizophrenics do or do not show a different performance from controls in this task. The less clear results could be due to this task being more difficult and less motivating than the Aging Faces task, specially for the groups selected for Experiment 2, who were older, less educated, and less used to computerized testing than the psychology students in Experiment 1. This possibility, however, predicts a decrease in precision (greater variable error) in the healthy participants in Experiment 2 vs. Experiment 1, but the opposite was actually the case: controls in Experiment 2 showed greater precision (smaller JND) than participants in Experiment 1 [1.13 vs. 1.46, respectively; *t*_(35)_ = −2.63, *p* = 0.01]. A motivational account is therefore ruled out. If finally schizophrenics are shown to have a dissociation between their performance in the Standard Time Bisection and Aging Faces tasks, other possibilities will need to be explored. The important point to emphasize here is that present results suggest that schizophrenics will either behave similarly in the two tasks (in both cases showing greater precision than controls) or they will show normal performance in the Standard Time Bisection task. Present data are not consistent with the possibility of a cross-over interaction between the two temporal tasks.

Finally, and centrally for the predictions of the common coding hypothesis, Experiment 2 found some correlations between the spatial task and each of the two temporal tasks. However, when the sample was expanded by including the participants in Experiment 1, any trace of significant correlations vanished. Even the two temporal tasks failed to correlate with each other. The overall implications of these findings are taken up in the following general discussion.

## General discussion

The present study revealed several interesting new findings, which are relevant to an understanding of the processing of space and time both in schizophrenics and in the general population, and which have implications for the common coding hypothesis. The most novel finding is the observation of greater precision in schizophrenics in both a spatial task (Line Bisection) and a temporal task (Aging Faces), as well as some marginal traces in the same direction in a second temporal task (Standard Time Bisection). Moreover, patients did not show the end bias that healthy controls did show in the Aging Faces temporal task. A second main finding was that all those tasks failed to correlate to each other, both across the spatial and temporal dimensions as well as within the temporal dimension alone.

We have found no previous reports of greater precision (reduced variable error) in bisection tasks in the literature on schizophrenia. Prior studies did not report variability in line bisection, so the present finding may have gone unnoticed. Regarding temporal tasks, prior studies most often showed worse precision (increased variable error) in schizophrenic patients (Allman and Meck, 2012; see Introduction). Moreover, extant evidence shows that schizophrenics do not always differ from controls in bias (constant error) in temporal tasks, but when they do, they usually show a stronger, instead of a reduced bias (see Introduction). Although the present findings are clear, the relatively small sample size in the patient and matched control groups suggest that they should be taken with caution and point to the need of additional research.

One limitation of the present study is the lack of an assessment of executive functions, as it has been shown that these functions are often deficitary in schizophrenia (see Orellana and Slachevsky, 2013, for a recent review). Moreover, there is independent evidence that the same brain structures that mediate executive functions play important roles in temporal cognition (Lewis and Miall, 2006). However, an executive deficit would predict worse performance in the schizophrenic group, contrary to present findings. Present results look more in line with published observations from a different clinical group, Tourette's syndrome. Patients with Tourette's syndrome are affected by uncontrollable tics, at least partially as a result of deranged activity in the dopamine-mediated circuit linking the basal ganglia and frontal lobes (McNaught and Mink, 2011). Thus, Tourette's syndrome may share a patophysiological substrate with schizophrenia. Paradoxically, Tourette's patients have shown better performance than healthy controls in cognitive control tasks (Jackson et al., 2007), and also in temporal reproduction tasks (Vicario et al., 2010). However, it would be premature to claim any clear relation between alterations in this neural substrate and improved temporal processing, as the evidence is still scant and the pattern complex (e.g., Vicario et al., 2010, reported better performance only in temporal reproduction task, but not in temporal discrimination, and only in intervals in the supra-second range).

All in all, the fact that both line bisection and temporal bisection in the Aging Faces task were clearly affected by schizophrenia, and that the Standard Time Bisection task also showed some marginal indications of effects in the same direction, suggests the existence of an underlying link and therefore constitute evidence in support of the common coding hypothesis of space and time. However, spatial and temporal tasks showed overall null correlations, both in bias and precision. Even the two temporal tasks failed to correlate to each other. Although present data are based on a relatively small sample size, they add to the evidence provided by Elvevåg et al. (2004), which is to our knowledge the only published study that has tested the processing of space and time in the same group of patients. These authors used absolute identification tasks, in which a series of stimuli (seven in their study) are presented and the participant learns to identify each one by means of a number corresponding to its location in the overall sequence (e.g., “this is tone number five”). Schizophrenic patients in this study showed a deficit in identifying the duration of tones (ranging from 333 to 2333 ms) and also in identifying letters within letter sequences (which is arguably a skill related to time processing). However, their discrimination of line lengths was unaffected, providing evidence for a dissociation between the magnitudes of space and time in schizophrenia. In the present study we did not find completely convincing dissociations between tasks at the group level, but the tasks failed to correlate over individuals. Related evidence against common coding of space and a different magnitude, number, in schizophrenia has been offered by Tian et al. (2011): their patient group showed a leftwards bias in line bisection but no bias in number bisection.

Present data and available related evidence thus support the conclusion that the common coding hypothesis may be stated in too general terms and it may need further qualification and development. Not all prothetic magnitudes are the same. Moreover, even single magnitudes such as space and time are not monolithical entities. There are different spaces and different times in the mind/brain. Spatial maps abound all over the brain (Silver and Kastner, 2009). Evidence suggests that the representation and processing of space differs depending on a variety of reference frames, including retinotopic (Silver and Kastner, 2009), object-centered (Olson, 2003), and peri-personal vs. extra-personal (Holmes and Spence, 2004). There may also be processing differences between the three spatial axes: lateral, vertical, and sagittal (Franklin and Tversky, 1990). Regarding time, there is convincing evidence that temporal intervals at sub-second and supra-second ranges may be processed by different mechanisms: In particular, short intervals up to the range of 1 s may be perceived directly, whereas longer intervals ranging from several seconds to minutes or longer units may require the implication of attentional and inferential mechanisms (Block, 1990; Zakay and Block, 1996). Moreover, it is well-established that temporal processing is the result of a brain-wide network incluiding the cerebellun and basal ganglia (see Grondin, 2010; Allman and Meck, 2012, for reviews). Magnitude processing in the parietal cortex is also part of a network that includes prefrontal regions, among others (Bueti et al., 2008). Lewis and Miall (2003) proposed a role for the parietal cortex only in the sub-second range. Future progress on the question of the representation of prothetic dimensions will sure benefit from a more nuanced view that will specify which aspects of which dimensions are likely candidates to be represented and served by a common mechanism, and where and how is such mechanism implemented in the brain.

### Conflict of interest statement

The authors declare that the research was conducted in the absence of any commercial or financial relationships that could be construed as a potential conflict of interest.

## Footnotes

^1^We thank Anne Giersch for pointing this index to us.
